# Facilitators and barriers to accessing reproductive health care for migrant beer promoters in Cambodia, Laos, Thailand and Vietnam: A mixed methods study

**DOI:** 10.1186/1744-8603-8-21

**Published:** 2012-07-02

**Authors:** Gail Webber, Denise Spitzer, Ratana Somrongthong, Truong Cong Dat, Somphone Kounnavongsa

**Affiliations:** 1 Bruyere Research Institute, Family Medicine, University of Ottawa, Ottawa, Canada; 2 Institute of Women’s Studies and Institute of Population Health, University of Ottawa, Ottawa, Canada; 3 College of Public Health Sciences,, Chulalongkorn University, Bangkok, Thailand; 4 Faculty of Public Health, Thai Binh Medical University, Thai Binh, Vietnam; 5 Graduate Office, National University of Laos, Vientiane, Laos

**Keywords:** Beer promoters, Migrants, Sexual health, Reproductive health, Access to health services, Southeast Asia, Cambodia, Laos, Thailand, Vietnam

## Abstract

**Background:**

The purpose of the research was to assess access to sexual and reproductive health services for migrant women who work as beer promoters. This mixed methods research was conducted in Phnom Penh, Cambodia, Bangkok, Thailand, Vientiane, Laos, and Hanoi, Vietnam during 2010 to 2011.

**Methods:**

Focus groups were held with beer promoters and separate focus groups or interviews with key informants to explore the factors affecting beer promoters’ access to health care institutions for reproductive health care. The findings of the focus groups were used to develop a survey for beer promoters. This survey was conducted in popular health institutions for these women in each of the four Asian cities.

**Results:**

Several common themes were evident. Work demands prevented beer promoters from accessing health care. Institutional factors affecting care included cost, location, environmental factors (e.g. waiting times, cleanliness and confidentiality) and service factors (e.g. staff attitudes, clinic hours, and availability of medications). Personal factors affecting access were shyness and fear, lack of knowledge, and support from family and friends.

The survey of the beer promoters confirmed that cost, location and both environmental and service factors impact on access to health care services for beer promoters. Many beer promoters are sexually active, and a significant proportion of those surveyed rely on sex work to supplement their income. Many also drink with their clients. Despite a few differences amongst the surveyed population, the findings were remarkably similar across the four research sites.

**Conclusions:**

Recommendations from the research include the provision of evening and weekend clinic hours to facilitate access, free or low cost clinics, and health insurance through employer or government plans which are easy to access for migrants. Other improvements that would facilitate the access of beer promoters to these services include increased funding to hire more staff (reducing waiting times) and to stock more needed medications, mobile clinics to come to the workplace or free transportation for beer promoters to the clinics, improved training to reduce health care provider stigma against beer promoters, and public education about the importance of reproductive health care, including preventative services.

## Introduction

Migration both across and within international borders has become a global phenomenon, impelled by contemporary globalization that is characterized by intensified global connections, and flexible labour processes [1,2]. The International Organization for Migration estimates that 214 million people have migrated across national borders, 49 percent of whom are women [3]. Women migrants are more likely to be engaged in poorly waged, casual labour than their male counterparts [1]. In addition to international migrants, there are many thousands who migrate within their countries, usually to larger cities. Individuals and families are pushed to migrate for factors such as war and natural disasters, but a significant number migrate for economic reasons, searching for viable options to support themselves and their families. This is the case for the young women in Southeast Asia who are the focus of this study. While they were raised in the countryside of their respective countries, these women moved to the cities for employment, recognizing that their options to make a living at home were few. Along with thousands of others, these migrant women work as beer promoters in restaurants, karaoke parlors, and beer shops in the large cities of Southeast Asia [4].

In recent years, many young women would have found work in manufacturing, most specifically in the lowest skilled and lowest salaried jobs located at the bottom of global supply chains [5]. Since the global financial crisis in 2009, however, the manufacturing sector has undergone a rapid decline. In Cambodia alone, where 20 percent of young women were employed in the garment industry, tens of thousands of workers have been laid off or have had their wages and working hours reduced [6,7]. Concomitantly, neo-liberal measures undertaken by national governments have resulted in the erosion of public health and social services and the implementation of a range of user fees, including user fees for health services, that together inhibit access by the most vulnerable, primarily women and children. As Desjardins [5,8] noted: “Workers in precarious employment, without economic and social entitlements, and without long-term career prospects or equipped with few skills are more vulnerable to risks of unexpected economic downswings, job and wage losses than other workers.” Indeed, as Petchesky succinctly summarized [[8], p. 140]]: “Women pay for the cumulative social deficits of global capitalism”.

It is in this context, that the young Southeast Asian women who are the focus of this study have left their rural birthplaces to seek employment in urban settings. Recognizing that their options to make a living at home were few and with the disappearance of many manufacturing jobs, these migrant women work as beer promoters in restaurants, karaoke parlors, and beer shops in the large cities of Southeast Asia. The purpose of this research is to assess the facilitators and barriers to reproductive health care services for this population of women using a mixed methods research design.

## Background

Beer promoters are employed by beer companies or local establishments to market particular brands of beer to customers. In 2004, the International Labour Organization (ILO) found that in Phnom Penh alone, 24 brands of beer were being promoted in this manner and over 4,000 women worked as beer promoters throughout the Cambodian capital [9]. Many companies contract young women based on their appearance, and most compel their workers to wear tight, revealing clothing that many find immodest [10]. Many beer promoters work in whole or in part on commission; therefore, keeping the customers satisfied is essential to maintaining their income. Resultantly, beer promoters contend with daily sexual harassment and with frequent demands to drink with their customers. In one survey, 15 percent of respondents reported being asked by their employer to engage in sexual relations with a customer [10]. In some Southeast Asian countries, beer promoters are categorized as “indirect sex workers,” and in all four countries included in our study, public discourse generally stigmatizes the work of beer promoters, relegating them to the category of “bad girl” [9,10].

While the prevalence of Human Immunodeficiency Virus and Acquired Immune Deficiency Syndrome (HIV/AIDS) is declining globally, it still remains a significant risk for beer promoters throughout South-East Asia. According to the Joint United Nations program on HIV/AIDS (UNAIDS) in Thailand the prevalence of HIV amongst indirect female sex workers, is 1.7 percent (2009 data). There is no HIV data on indirect sex workers for the other three countries, however, in Cambodia, despite declines in the HIV prevalence generally, the infection remained at over 14 percent amongst female sex workers in 2006, while in Vietnam where the epidemic is currently occurring mostly through sexual transmission, the prevalence amongst female sex workers is 3.2 percent (2009 data) [11]. In Laos the HIV epidemic is fortunately limited, however, the population is young: 60 percent of the total population is below 25 years of age [12]. With rising rates of sexual activity amongst youth, there are significant risks of HIV spread.

Access of beer promoters to sexual and reproductive health care services was deemed an important priority at an October 2009 research meeting of academics, government and non-governmental organization (NGO) staff, beer industry representatives, and beer promoters from Cambodia, Laos, Thailand, and Vietnam and was subsequently confirmed by focus groups with beer promoters [13]. Lack of time to access services, cost and availability of services, health care provider stigma, and shyness of the beer promoters were all factors impacting on access to reproductive health care services for these women.

Gaining access to primary health care services, including sexual and reproductive health care, is complex. Wellstood and colleagues [14] have written how access to primary health care is dependent on individual characteristics of the user such as income, age, gender, and level of need, as well as system characteristics and the policy environment. In their analysis, economic factors, geography, availability of services, and socio-cultural issues are all key elements of access. While Wellstood’s research took place within the Canadian context, there is evidence from elsewhere that that this conceptual framework applies to wider populations. For example, researchers have found that factors at the individual, relationship, community, and structural level (both policy and cultural), impact access to HIV prevention for migrant women [15]. Employing this conceptual framework, our research has attempted to identify the individual, system, and policy factors impacting access to sexual and reproductive health care services for migrant beer promoters in Cambodia, Laos, Thailand, and Vietnam.

## Methods

### Research team

Our research team emerged from a research agenda meeting held in October 2009, in Phnom Penh, Cambodia. The research team consisted of the Canadian principal investigator (Webber) and co-investigator (Spitzer) in addition to a co-investigator from each of the four research sites (Cambodia: Bunnak; Laos: Kounnavongsa; Thailand: Somrongthong; Vietnam: Dat) and a research co-ordinator. Each Asian co-investigator led a team with a country research manager and two research assistants. The country research manager was responsible for helping organize the research locally and for supervision of the two research assistants. In all countries, at least one of the research assistants had experience working as a beer promoter and all research assistants were female. This was felt to be important as the beer promoter research participants were likely to feel more comfortable speaking with other women who shared the status and challenges stemming from their work as beer promoters about matters of their sexual and reproductive health. Our research co-ordinator was responsible for liaising with all teams on the research process and data collection, and for organizing the training meeting and final knowledge translation meetings.

### Research sites

The research was conducted in the four capital cities of the participating countries: Phnom Penh, Cambodia; Vientiane, Laos; Bangkok, Thailand; and Hanoi, Vietnam.

### Research design

This research project was a two-phase participatory mixed methods study. The first phase consisted of qualitative focus groups with beer promoters and key informants while the second phase was a survey of beer promoters and case studies of health care institutions. The case studies are published in a separate paper [16]. This research design was an exploratory sequential design, in which the qualitative first phase was used to explore the issues and inform the development of the quantitative survey tool used in the second phase [17].

Ethics approval for both phases of the research was obtained in Canada, in addition to individual ethics boards in Cambodia, Laos, Thailand and Vietnam. As an international research team, we were cognizant of the diverse ethical issues of researcher identity in this global health research [18]. In accordance with feminist and participatory approaches to research [19,20], we hired local beer promoters whom we trained as research assistants to both promote skills transfer and to narrow the gap between research participants and data collectors.

In the first phase of the research, focus groups were held with beer promoters and separate focus groups were held with key informants. Focus groups are particularly useful as a data collection strategy to explore research participants’ views on a subject [21]. The purpose of these focus groups was to understand the barriers and facilitators of access for beer promoters to sexual and reproductive health care. The focus group questions addressed the individual, family, institutional, community, and policy factors that may impact access to sexual and reproductive health for beer promoters. A minimum of four focus groups each consisting of about eight to ten beer promoters were recruited through snowball sampling. Focus group participants were paid $10 US for their participation. This amount was deemed appropriate by local researchers as a stipend to replace lost wages, and not so large as to be coercive [22]. In addition, each site hosted a minimum of four focus groups of key informants. Key informants included health care providers working in institutions providing sexual and reproductive health care to beer promoters in the government, non-governmental, and private sectors in addition to policy makers in the government and senior non- governmental organization staff. The recruitment of key informants was purposive, with the goal of having a balance of health care providers and policy makers. If it was not possible to schedule focus groups with important key informants due to either time constraints or their personal preference, individual interviews were conducted instead.

All focus groups were held in the local language and were recorded. Where recording was not permitted detailed notes were taken. The recordings or notes were transcribed by the research assistants and translated into English. All names and identifying information were removed from the transcripts before translation. Translation was done by the local research team members who were present during the focus groups to minimize errors [23]. The local co-investigators provided the English transcripts of the focus groups to the Canadian researchers. The two Canadian investigators analyzed the original transcripts using N-Vivo version 8, qualitative software for both content and thematic analysis.

In phase two, the draft beer promoter survey was modified based on the findings from the first phase of the study [21]. All co-investigators reviewed and approved the final draft. The English version was translated into Khmer, Laotian, Thai and Vietnamese and then back-translated into English. All back translations were reviewed by the Principal Investigator and revisions were made by the country research teams. The goal was to survey 100 beer promoters by each of the four country research teams. The sample size was determined by an estimated population of several thousand beer promoters within each capital [13], and a desired precision of at least 10 percent [24]. The survey questions focused on demographic information about the beer promoter, factors affecting their choice of health care institution, their health care experiences and their behaviors. Surveys were conducted at popular health care institutions frequented by beer promoters. Beer promoters were paid $5 US for their participation in the survey. In addition to the survey, each country research team conducted the case study of the health care institutions used for the survey. The case studies focussed on the services offered at the institution, provider training and skills, and facilities at the institution.

The country research teams entered all the data from the surveys into an Excel spreadsheet. Template Excel coding tables were provided to each country co-investigator by the principal investigator. The Canadian PI was responsible for the final statistical analysis of the data. Using SPSS, data were analyzed for descriptive similarities and differences between countries and for trends in findings. The completed case study surveys were returned to the Canadian PI and a summary of the results was prepared for comparisons between countries.

### Consent

Written consent was obtained from all research participants for anonymous use of their quotations in publications.

## Results

The phase one results were collected from July 2010 to November 2010. The number of focus groups with beer promoters and focus groups or interviews with key informants for each country are listed in Table 1.

**Table 1:** Phase 1 Research Participants

**Country**	**Beer Promoter Focus Groups**	**Key Informant Focus Groups/Interviews**
Cambodia	Four focus groups	Eleven interviews or small focus groups
	TOTAL 36 beer promoters	TOTAL 19 Key Informants
Laos	Five focus groups	Five interviews
	TOTAL 43 beer promoters	TOTAL 5 Key Informants
Thailand	Five focus groups	Four interviews and One Focus group
	TOTAL 47 beer promoters	TOTAL 11 Key Informants
Vietnam	Four focus groups	Five interviews
		Two Focus Groups
	TOTAL 34 beer promoters	TOTAL 20 Key Informants

The phase 2 survey of beer promoters was conducted during February to April 2011 in each of the four research locations. The types of institutions used to conduct the survey and the number of beer promoters surveyed in each institution are listed below in Table 2. The total number of beer promoters surveyed was 390.

**Table 2:** Phase 2 Research Participants

**Research Site**	**Type of Institution**	**Number of Beer Promoters Surveyed: Per Institution/Country Total**	
Phnom Penh, Cambodia	Non Governmental Organization	60	90
	Non Governmental Organization	30	
Vientiane, Laos	Government Health Centre	49	100
	Non Governmental Organization	51	
Bangkok, Thailand	Government Health Center	50	100
	Government Hospital	50	
Hanoi, Vietnam	Non Governmental Organization	100	100

The results will be presented in the following manner: the demographic description of the survey data will be followed by a summary of the health behaviors of the beer promoters. Thereafter we include a discussion of beer promoters’ experiences with, and their preferences for, health care institutions. The quantitative results will be illustrated by qualitative examples where the results concur, and with contrasting examples where they differ. While the qualitative focus group data were collected before the surveys were conducted, we have chosen to present and discuss the results together in order to avoid redundancy and for purposes of comparison of both forms of data. Differences between countries will also be illustrated, though the numbers were not large enough to demonstrate statistical significance.

### Summary of demographics of survey population

The demographic details of the beer promoters who participated in the survey are illustrated in supplementary file Table 3.

**Table 3:** Demographics of Beer Promoters Surveyed

**Item**	**Category**	**Cambodia n = 90 (%)**	**Laos n = 100 (%)**	**Thailand n = 100**	**Vietnam n = 100**	**Total n = 390 (%)**
1. Age (years):	17–19	10 (11.1)	29	8	15	62 (15.9)
	20–24	29 (32.2)	57	59	40	185 (47.4)
	25–29	19 (21.1)	11	29	26	85 (21.2)
	30–39	28 (31.1)	3	4	19	54 (13.8)
	40–47	4 (4.4)	0	0	0	4 (1.0)
	Mean Age	26.8	21.5	23.6	25.0	24.2
2. Marital status: * * 5 missing values for Laos	Married	29 (32.2)	3 (3.2)	1	21	54 (14.0)
	Never married	19 (21.1)	62 (65.3)	80	44	205 (53.2)
	Cohabiting	10 (11.1)	2 (2.1)	0	16	28 (7.3)
	Divorced	7 (7.8)	28 (29.5)	0	15	50 (13.0)
	Widowed	7 (7.8)	0	1	2	10 (2.6)
	Separated	18 (20)	0	18	2	38 (9.9)
3. Number of Children:	0	32 (35.6)	73	85	60	250 (64.1)
	1	28 (31.1)	17	9	23	77 (19.7)
	2	22 (24.4)	7	4	15	48 (12.3)
	3 or more	8 (8.9)	3	2	2	15 (3.8)
4. Years since migration:* 5 missing values for Laos	Mean Years	7.2	1.9	4.3	3.2	4.1
5. Years worked as beer promoter	Mean Years	3.3	1.1	1.8	1.8	1.9
6. Number working full-time/part-time:* *6 missing values for Laos	Full-time	54 (60.0)	73 (77.7)	83	63	273 (71.1)
	Part-time	36 (40.0)	21 (22.3)	17	37	111 (28.9)
7. Locations Worked:* May total > 100 as could indicate more than 1 response	Karaoke	15 (16.7)	4	8	74	101 (25.9)
	Restaurant	52 (57.8)	4	58	18	132 (33.8)
	Beer Shop	23 (25.6)	89	32	24	168 (43.1)
	Other	0	0	2	2	4 (1.0)
8. Number of family members supported:* * 10 missing values for Laos	0	1 (1.1)	19 (21.1)	54	27	101 (26.8)
	1	2 (2.2)	5 (5.6)	9	26	42 (11.1)
	2	17 (18.9)	15 (16.7)	17	22	71 (18.7)
	3	26 (28.9)	10 (11.1)	7	8	51 (13.4)
	4	15 (16.7)	8 (8.9)	5	10	38 (10.0)
	5 or more	29 (32.2)	33 (36.7)	8	7	77 (20.2)
9. Number with Health Insurance:		24 (26.7)	8	97	7	139 (35.6)
10. Provider of Health Insurance:	Government	0	3	27	5	35 (9.0)
	Employer	15 (16.7)	5	62	2	84 (21.5)
	Other	9 (10.0)	0	11	0	20 (5.1)
11. Institutions used for reproductive health care: * 1 missing value for Laos for private clinic	Government Health Centre	17 (18.9)	52	50	26	145 (37.2)
	Private Clinic*	0	46 (46.5)	5	47	98 (25.1)
	Pharmacy	0	77	20	54	151 (38.7)
	NGO Clinic	72 (80.0)	9	0	80	161 (41.3)
	Government Hospital	1 (1.1)	62	55	21	139 (35.6)
	Private Hospital	0	5	58	26	69 (17.7)
	Other	0	0	0	0	0
	No place	0	0	2	0	2 (0.5)
12. Reason for Visit Today:	Vaginal Discharge	54 (60.0)	85	39	64	242 (62.1)
	Contraception	8 (8.9)	47	4	13	72 (18.5)
	Menstrual Problems	5 (5.6)	58	33	31	127 (32.6)
	Pregnancy	3 (3.3)	22	0	15	40 (10.3)
	Abortion	3 (3.3)	38	0	15	56 (14.4)
	Testing for Reproductive Health Problems	15 (16.7)	28	1	14	58 (14.9)
	Other reproductive health problems	1 (1.1)	0	8	0	9 (2.3)
	Other non-reproductive health problems	1 (1.1)	0	3	10	14 (3.6)
	No answer given	0	0	32	4	36 (9.2)

Of the women surveyed, well over half were under 25 (63.3%). The mean age of the participants was 24.2 years. There were some differences between the demographic variables in the four countries. The Cambodia and Vietnamese participants were older than the Thai and Lao participants (one third of the Cambodian group was 30 or older). Similarly, the majority of Lao and Thai participants had never married and were childless while only one third of the Cambodian participants were without children. Forty percent of the Vietnamese participants had one or more children. Not surprisingly as they were younger on average, the Lao beer promoters had migrated most recently (mean 1.9 years) and worked as beer promoters for the shortest time (mean 1.1 years), while the Cambodians had migrated earlier (mean 7.2 years) and worked the longest as beer promoters (mean 3.3 years). The majority of the women surveyed from all countries worked as full-time beer promoters (71.1%). The most common locations worked varied by country: karaoke was the most frequently cited location for the Vietnamese participants (74%), while most Thai and Cambodian beer promoters worked in restaurants (58% and 57.8% respectively). The beer shop was the common location for the Lao beer promoters who were surveyed (89%).

Almost three quarters of the beer promoters were financially supporting at least one family member, while over 20% were supporting five or more members. This was less of a trend for the Thai beer promoters: over half of these women did not support any family members. With the exception of Thailand, most beer promoters lacked health insurance. Only a quarter had some insurance in Cambodia, while over 90% lacked health insurance in both Laos and Vietnam. In Thailand, where 97% of respondents had insurance, employers were primarily responsible for its provision.

The beer promoters surveyed accessed reproductive health care in a variety of locations as noted in supplementary file Table 3. The most common reason for accessing service was for vaginal discharge, followed by menstrual problems, the need for contraception, testing for reproductive health problems, abortion services and prenatal care.

### Summary of health behaviors

The health behaviors of the beer promoters in the survey are listed in supplementary file Table 4. The women commonly reported having sex outside of their primary relationship. While only 18.2% admitted to frequent sexual relations outside of their primary relationship (more than once per month), a further 34.1% stated they had extramarital sex at least once per year. These sexual activities were common amongst all four nationalities of beer promoters.

**Table 4:** Health Behaviors of Beer Promoters

**Item**	**Response**	**Cambodia n = 90 (%)**	**Laos n = 100**	**Thailand n = 100**	**Vietnam n = 100 (%)**	**Total (%)**
43. Sex outside of marriage:	1	30 (33.3)	15	41	17	103 (26.4)
	2	13 (14.4)	36	20	14	83 (21.3)
	3	35 (38.9)	31	26	41	133 (34.1)
	4	12 (13.3)	18	13	28	71 (18.2)
44. Sex with clients:	1	32 (35.6)	7	78	22	139 (35.6)
	2	18 (20)	14	12	21	65 (16.7)
	3	32 (35.6)	39	6	40	117 (30.0)
	4	8 (8.9)	40	4	17	69 (17.7)
45. Money for sex: * 1 missing value for Cambodia	1	28 (31.4)	7	82	24	141 (36.2)
	2	16 (18.0)	3	7	17	43 (11.1)
	3	35 (39.3)	20	11	32	98 (25.2)
	4	10 (11.2)	70	0	27	107 (27.5)
46. Drunk beer with clients:	1	18 (20)	4	58	5	85 (21.8)
	2	9 (10)	5	25	18	57 (14.6)
	3	37 (41.1)	42	14	39	132 (33.8)
	4	26 (28.9)	49	3	38	116 (29.7)
47. Suspected or known sexually transmitted infection:	1	39 (43.3)	17	86	33	175 (44.9)
	2	14 (15.6)	25	10	29	78 (20.0)
	3	23 (25.6)	46	4	35	108 (27.7)
	4	14 (15.6)	12	0	3	29 (7.4)
48. Used injection drugs:	1	68 (75.6)	90	87	96	341 (87.4)
	2	5 (5.6)	7	7	2	21 (5.4)
	3	9 (10)	3	6	1	19 (4.9)
	4	8 (8.9)	0	0	1	9 (2.3)
49. Had an abortion: *Problem with category - should not be 4.	1	39 (43.3)	40	89	48	216 (55.4)
	2	17 (18.9)	49	7	39	112 (28.7)
	3	21 (23.3)	10	4	13	48 (12.3)
	4*	13 (14.4)*	1*	0	0	14 (3.6)
50. Sex with men for money to supplement income:	1	36 (40)	20	89	24	169 (43.3)
	2	15 (16.7)	13	8	19	55 (14.1)
	3	29 (32.2)	39	3	36	107 (27.4)
	4	10 (11.1)	28	0	21	59 (15.1)
51. Drunk from drinking at work:	1	21 (23.3)	13	77	41	152 (39.0)
	2	23 (25.6)	11	16	30	80 (20.5)
	3	29 (32.2)	48	6	26	109 (27.9)
	4	17 (18.9)	28	1	3	49 (12.6)
52. Beer promotion work provide enough money * 2 missing values for Vietnam	1	32 (35.6)	8	28	15 (15.3)	83 (21.4)
	2	21 (23.3)	11	22	16 (16.3)	70 (18.0)
	3	27 (30)	41	19	41 (41.8)	128 (33.0)
	4	10 (11.1)	40	31	26 (26.5)	107 (27.6)

1 = never, 2 = rarely (less than once per year), 3 = sometimes (at least once per year but no more than once per month), 4 = often (more than once per month).

The beer promoters were questioned about sex work on 3 different items “sex with clients” (Item 44), “receiving money for sex” (Item 45), and “sex with men to supplement their income” (Item 50). Over one third denied any sex work on these three items (35.6%, 36.2% and 43.3% respectively). The frequency of women who admitted that they “sometimes” (at least yearly) or “often” (at least monthly) engaged in sex work was generally higher: responses of “sometimes” or “often” on the three items addressing this issue were 47.7%, 52.7% and 42.5% respectively. Sex work was most common in beer promoters from Laos and Vietnam, and least common amongst the Thai beer promoters.

More than half the survey participants from Cambodia, Laos and Vietnam and 11% in Thailand had had an abortion at some point in their lives. (Note that in the Cambodian and Laotian surveys the response category of 4 or “often” was mistakenly included in the survey although it should not apply to this question).

The beer promoters surveyed were questioned about drinking beer with clients (item 46) and getting drunk at work (item 51). Drinking beer with clients was common amongst all beer promoters except those from Thailand. Women reported “sometimes” or “often” drinking beer with clients: 70% in Cambodia, 91% in Laos, 77% in Vietnam and only 17% in Thailand. Similarly, reports of getting drunk at work “sometimes” or “often” were highest in Laos (76%) and Cambodia (51.1%) followed by Vietnam (29%) and Thailand (7%).

About one third of the beer promoters reported that they “sometimes” or “often” knew or suspected that they had a sexually transmitted infection, though again this proportion varied by country: Laos 58%, Cambodia 41.2%, Vietnam 38% and Thailand only 4%.

Injection drug use was uncommon in all countries: only 7.2% of women admitted to “sometimes” or “often” using injection drugs and its use was most common in the Cambodian cohort (18.9% of women states “sometimes” or “often” using drugs).

When questioned if their work as beer promoters provided enough money to live on about one fifth stated that it “never” did though again the responses varied by country from Cambodia (35.6%) and Thailand (28%) to Vietnam (15.3%) and Laos (8%).

### Factors affecting choice of health care institutions

Table 5 (supplementary file) documents the beer promoters responses to what issues are important to them in their choice of health care institution for sexual and reproductive health care.

**Table 5:** Choice of Health Care Institution

**Item**	**Response**	**Cambodia n = 90 (%)**	**Laos n = 100 (%)**	**Thailand n = 100**	**Vietnam n = 100**	**Total (%)**
13. Cost of Services is very important:	1 & 2	70 (77.8)	85	58	67	280 (71.8)
	3	13 (14.4)	12	25	18	68 (17.4)
	4 & 5	7 (7.8)	3	17	15	42 (10.8)
14. Location of services is very important:	1 & 2	74 (82.2)	88	58	62	282 (72.3)
	3	8 (8.9)	7	26	14	55 (14.1)
	4 & 5	8 (8.9)	5	16	24	53 (13.6)
15. Friendliness of providers is important: * 1 missing value for Laos	1 & 2	82 (91.2)	84 (84.8)	71	94	231 (85.1)
	3	7 (7.8)	15 (15.2)	22	2	46 (11.8)
	4 & 5	1 (1.1)	0	7	4	12 (3.1)
16. I am likely to rely on recommendations of friends:	1 & 2	69 (76.7)	43	52	68	232 (59.5)
	3	12 (13.3)	40	38	16	106 (27.2)
	4 & 5	9 (10.0)	17	10	16	52 (13.3)
17. If long waits, I go elsewhere: * 1 missing value for Laos	1 & 2	40 (44.5)	33 (33.3)	58	43	174 (44.7)
	3	18 (20.0)	31 (31.3)	25	27	101 (26.0)
	4 & 5	32 (35.5)	35 (35.4)	17	30	114 (29.3)
18. I avoid care as I am shy:	1 & 2	22 (24.4)	34	46	23	125 (32.1)
	3	9 (10.0)	10	32	14	65 (16.7)
	4 & 5	59 (65.6)	56	22	63	200 (51.3)
19. I prefer a female health care provider for reproductive exams:	1 & 2	76 (84.4)	71	56	72	275 (70.5)
	3	3 (3.3)	17	35	12	67 (17.2)
	4 & 5	11 (12.2)	12	9	16	48 (12.3)
20. Money/Gifts for provider improve service:	1 & 2	35 (38.9)	4	49	38	126 (32.3)
	3	6 (6.7)	37	29	25	97 (24.9)
	4 & 5	49 (54.4)	59	22	37	167 (42.8)
21. I fear examinations thus I avoid care:	1 & 2	24 (26.7)	18	32	14	88 (22.6)
	3	11 (12.2)	16	38	18	83 (21.3)
	4 & 5	55 (61.1)	66	30	68	219 (56.2)
22. I cannot afford health care at institution I prefer:	1 & 2	44 (48.9)	52	39	35	170 (43.6)
	3	13 (14.4)	30	31	30	104 (26.7)
	4 & 5	33 (36.7)	18	30	35	116 (29.7)
23. I cannot get time off work to get health care:	1 & 2	31 (34.4)	25	45	45	146 (37.4)
	3	10 (11.1)	37	26	17	90 (23.1)
	4 & 5	49 (54.4)	38	29	38	154 (39.5)
24. Cost of Transportation is key factor:	1 & 2	42 (46.7)	74	44	32	192 (49.2)
	3	13 (14.4)	16	37	25	91 (23.3)
	4 & 5	35 (38.9)	10	19	38	107 (27.4)
25. Confidentiality is very important: * 1 missing value for Laos	1 & 2	76 (84.4)	88 (88.9)	75	89	328 (84.3)
	3	4 (4.4)	8 (8.1)	21	7	40 (10.3)
	4 & 5	10 (11.1)	3 (3.0)	4	4	21 (5.4)
26. I choose based on skills of health care providers: *1 missing value for Laos	1 & 2	83 (92.2)	80 (80.1)	69	95	327 (84.1)
	3	5 (5.6)	19 (19.2)	25	4	53 (13.6)
	4 & 5	2 (2.2)	0	6	1	9 (2.3)
27. I choose based on caring attitudes of health care providers: *1 missing value for Laos	1 & 2	84 (93.3)	81 (81.8)	80	90	335 (86.1)
	3	4 (4.4)	16 (16.2)	17	2	39 (10.0)
	4 & 5	2 (2.2)	2 (2.0)	3	8	15 (3.9)
28. In past I was treated badly by health care providers:	1 & 2	19 (21.1)	6	40	28	93 (23.8)
	3	9 (10.0)	24	38	18	89 (22.8)
	4 & 5	62 (68.9)	70	22	54	208 (53.3)
29. I don’t mind waiting more than 2 hours for care:	1 & 2	75 (83.3)	41	25	37	168 (43.1)
	3	14 (15.6)	30	42	23	109 (27.9)
	4 & 5	11 (12.2)	29	33	40	113 (28.9)
30. Cleanliness of Institution an important factor:	1 & 2	87 (96.7)	73	73	95	328 (84.1)
	3	2 (2.2)	20	23	2	47 (12.1)
	4 & 5	1 (1.1)	7	4	3	15 (3.8)
31. Care for non-reproductive health problems:	1 & 2	81 (90.0)	84	61	74	290 (74.4)
	3	6 (6.7)	13	29	11	59 (15.1)
	4 & 5	3 (3.3)	3	10	25	41 (10.5)
32. Health care provider give explanations:	1 & 2	85 (94.4)	85	65	95	330 (84.6)
	3	5 (5.6)	12	24	1	42 (10.8)
	4 & 5	0	3	11	4	18 (4.6)
33. I think male providers are more skilled than female:	1 & 2	44 (48.9)	25	22	19	110 (28.2)
	3	19 (21.1)	43	50	31	143 (36.7)
	4 & 5	27 (30.0)	31	28	50	137 (35.1)

1 = strongly agree, 2 = agree, 3 = neither agree nor disagree, 4 = disagree, 5 = strongly disagree.

### Barriers and facilitators to accessing sexual and reproductive health services

The potential barriers or facilitators to accessing sexual and reproductive health care services for beer promoters in these four Southeast Asian capital cities can be categorized under three major conceptual structures: institutional factors, work factors, and personal factors.

#### Institutional factors

There were several key factors that were common barriers preventing access for beer promoters to the institutions providing sexual and reproductive health care services in these four Asian capitals. These include financial barriers, location/ transportation issues, the environment of the institution, and service factors.

#### Financial barriers and health care insurance

Cost of the health care services was an important issue for these beer promoters as their financial resources were often stretched very thin. In the survey, 71.8 percent agreed or strongly agreed that cost was a very important factor in choice of health care institution (item 13). One third to one half could not afford to go to the health care institution that they preferred (item 22). The Cambodian and Vietnamese beer promoters quoted below reflect on the challenges they had balancing their own health with the financial needs of their families. Purchasing health care services meant giving up funds for other important requirements such as food, rent and family support. As a result, they would often avoid costly health care services.

If I go to [NGO name], I would like to use the Norplant and they wrote on the board that cost $50. And I do not have ability to pay for that because it is very expensive. For the beer seller like me, I have only the money to pay for the house rent, eating, send to my children and spend on other things. I do not have money for that. (Cambodia, FG BP 1)

You know that we come here to work to earn money and send money home, if we go to doctors, we don't have money for the time we go and we also have to spend money for doctors… Only when I have serious disease, I go to doctors because going to doctors takes much money. (Vietnam, FG BP 3)

In addition to regular fees of some of the health care institutions, this beer promoter from Laos noted that to get good service, sometimes gifts need to be provided to the doctors and nurses. As a poor beer promoter from the countryside, she was both unaware of this practice and unable to afford it.

Big city people know what to do when they go to the hospital. They always give doctors/nurses some gifts and they are looked after very well. People from countryside don’t quite know the system and they also don’t have extra money so they don’t know what to do. (Laos, FG BP 3)

The beer promoter from Laos quoted above was somewhat unique in her experience among her cohorts from her country. Only 4 percent of Laotian beer promoters agreed or strongly agreed that money or gifts improve the service of health care providers (item 20), though overall about one third of the beer promoters did agree with this statement.

Financial issues underpin the hierarchy of resort [25], the progression of strategies used to resolve their health problems, most commonly shared by respondents. For most, the first step was the local pharmacy where they can obtain inexpensive medication without a physician’s prescription. If the medication did not adequately address the problem, the beer promoter resorted to the next, generally more expensive and more technologically sophisticated service, continuing until her needs have been satisfied. As one Laotian informant shared:

First, I go to the drugstore and buy medicine without prescription followed by using clinic service and finally going to the hospital if the health problem is still there. (Laos, FG BP 2)

As noted earlier, with the exception of those from Thailand, only about one third of the beer promoters had access to health insurance. For a few Cambodian and Vietnamese women, the employer provided this benefit: For my company, they have insurance. If we are sick, but not serious condition, we can go to the company clinic. If we are serious, we can go outside and have a receipt for them. (Cambodia, FG BP 4). If you work for long time at a permanent job like us, you will have contract and you will have health insurance. (Vietnam, FG BP 4)

The majority of beer promoters in the study, however, had no health insurance either because it was not provided by their employer or because they were part-time employees and it was not available to them. Government insurance was accessible for some Thai and Vietnamese beer promoters, but as migrants to the city it was not easy for them to use this insurance. Public health insurance schemes in those two countries are linked to service provision in residents’ natal communities, limiting its utility under conditions of migration.

Interviewer: Do you know about voluntary health insurance? Patients with voluntary health insurance only pay 5% to 10% of their hospital fees.

Respondent: Of course we know, but it requires a permanent address here while all of us are migrants. We only can buy health insurance at our hometown with a permanent address and we have to register at one hospital near our home to be examined. (Vietnam, FG BP 3)

For the beer promoters with health insurance, sometimes the health care institutions that were available to them were not desirable: The social security hospital is not providing good services, and doctors don’t care about us, long waiting. Actually we selected the clinic that is close to our home, convenient and clean. (Thailand, FG BP 3)

The Vietnamese key informant quoted below noted that while the government had policies to provide health care insurance, many beer promoters lacked labour contracts and thus were not protected by these laws and policies.

Most girls doing massage services, or working as beer promoters do not have labor contracts so they do not have social insurance or health insurance. Therefore, they do not have the opportunity to be examined in health centers or hospitals although our nation has laws to require these units to send their staff to health centers and have periodic health examinations. Besides, our nation also has laws to require heads of companies or restaurants, etc. to buy health insurance and social insurance for their staff but they usually don't buy so if beer promoters want to have their health checked, they must pay money themselves. (Vietnam, KI 2)

Thus, the cost of health care prevents beer promoters from accessing reproductive health care services. While health insurance would improve access, the complexities of government policies and lack of enforcement makes it difficult for poor beer promoters from the countryside to access health care insurance even if it exists.

#### Location/transportation factors

The location of the health care institution is often a key factor to whether or not beer promoters decide to access the services. In the survey 72.3 percent stated that location was important in their choice of health care institution, and the majority of beer promoters from all four countries held this opinion (item 14). In addition, almost a half of all the beer promoters and 74 percent of the Laotian beer promoters in particular agreed or strongly agreed that the cost of transportation was a key factor in accessing health care (item 24). Many beer promoters chose to seek care in institutions that are close to home as they are hesitant to spend money on transportation. This Thai beer promoter reflects on her priorities in choosing where to obtain health care:

Similar to the others, I usually go to a clinic because it is close to home, convenient and has quick services. (Thailand, FG BP 1)

Location and lack of transportation are an issue for this beer promoter in Laos. She felt she was better off in the countryside.

I don’t have a vehicle so it is very difficult for me to go to the health care provider. I am also shy to discuss my health problems with other people. I don’t have permanent address or even an identity card. It was not this difficult in my home town. (Laos, FG BP 3)

While convenience of location was a factor for beer promoters from all countries, some beer promoters were fortunate enough to be serviced by clinics that provided free or subsidized transportation. This transportation facilitated care and health education for some women:

That hospital always comes and collects all of us to receive the treatment and to understand about what we can do to take care of our health. (Cambodia FG BP 3)

#### Environmental factors

In addition to financial barriers and location/transportation issues, the environment of the clinic had an impact on accessibility to reproductive health care services. In particular, the beer promoters needed quick appointments, and often avoided health care institutions with long waits. In the survey, 44.7 percent of the beer promoters agreed that they would go elsewhere if there was a long wait (item 17). This varied from one third of the group in Laos to over a half of the Thai beer promoters. When questioned again if they would be willing to wait more than two hours for an appointment (item 29), 43.1 percent stated they would not mind (85.5 percent for Cambodia). Waiting was a common experience for beer promoters. Generally, the private health clinics had shorter waiting times than the government hospitals.

The government hospital is very crowded and it has a long waiting time; almost 50 people before me. (Thailand, FG BP 4).

Another key factor for choice of institution was the perceived cleanliness: eighty four percent of the beer promoters agreed or strongly agreed that cleanliness affected their choice (item 30). Some beer promoters preferred the private clinics as they were thought to be cleaner. This Vietnamese key informant summarized the strengths of the private clinics for beer promoters: Private clinics are open in order to earn money from patients so they usually meet all demands of patients such as fast, convenient, clean, friendly doctors and nurses. (Vietnam, FG KI 2)

Confidentiality was considered “very important” for 84.4 percent of surveyed beer promoters; only 5.4% disagreed or strongly disagreed (item 25). This Vietnamese beer promoter felt that the shorter waiting time and increased confidentiality of the private clinic was worth paying for: In private clinics we have to pay more money, but we don't have to wait for a long time and information is kept in secret, nobody knows who we are. (Vietnam, FG BP 4)

Not all agreed about the benefits of private clinics. This beer promoter from Laos preferred the quality of service and anonymity of the hospital: I am not confident to go to clinics. It seems okay during the medication, but the symptoms come back later. I also feel shy to tell doctor at clinic my problem person to person. Without witness, who will be responsible if something goes wrong? I am also afraid that I will meet the doctor from clinic in public and of course I will be recognized with my health problem. At hospitals, there are many patients and I am quite positive that the doctor will not remember my face as he/she sees many sick people every day. (Laos, FG BP 3)

In summary, the environment of the health care institution affects access of beer promoters to reproductive health care services. In particular, waiting times, cleanliness and confidentiality are important factors to these women.

#### Service factors

There are several factors related to the service provided by the health care institution that have an important impact on access to sexual and reproductive health care services for these women. One of the most significant was the friendliness and attitudes of the health care providers. More than 85 percent of the beer promoters felt that friendliness of the providers was an important issue (item 15) and few beer promoters disagreed (3.1percent). Over 85 chose the health care institution based on the caring attitudes of the providers (item 27). Health care provider skills were also perceived as important by over 84 percent (item 26). Health care provider explanations were deemed important by close to 85 percent (item 32). Female health care providers were preferred for reproductive health exams by over 70 percent (item 19), though this varied by country with Cambodian beer promoters having the strongest preference (84.4 percent), and Thai the least (56 percent). Conversely, Cambodian beer promoters were more likely to agree that male providers were more skilled than female providers, than beer promoters from other countries (item 33).

A minority of beer promoters agreed that they had been treated badly by health care providers in the past (item 28), though this varied from six percent in Laos to 40 percent in Thailand. It was not unusual to be shouted at, or treated with disrespect by health care providers. Some of the beer promoters perceived this was worse if they were dressed in their working clothes, as the health care providers stigmatized them for their work and their lower social status. Below are several examples of what women experienced:

Respondent 1: Each hospital provides a different quality of service, but how doctors react toward patients are pretty much the same. Some are very kind while some others seem to be very impolite.

Respondent 2: Doctors/nurses will talk to rich people very nicely. Those who are poor are ignored. (Laos, BP FG 2)

Attitude [of health care providers] may affect my decision [about choice of hospital]. They did not take good care of me. (Thailand, FG BP 4)

I have to say that we are beer promoters, we work in this environment, but we are also people. We also know how to hate, love, be angry, etc. And we get diseases or not, we are still people. Some doctors, they say and behave to us very softly and gently so that even when I don’t want to be checked, I let them examine me. But there are also some doctors, truthfully, we can’t smell them. They shout at us “examine or go home” or “wait out there”. In this situation, even if I have disease, I will not seek examination or treatment. (Vietnam, FG BP 2)

The doctors at [Name of Institution] said bad words. The doctor said that when we have sexual relationship, why don’t we call them to see? (Cambodia, FG BP 4)

This last example demonstrates the deep stigma some providers have against women who may be involved in sex work. This stigma was not unique to Cambodian health care providers. The Vietnamese key informant quoted below described how hospital training sessions are organized to improve communication and staff attitudes, but are not always effective.

I know some hospitals organize training sessions for their staff about communication ways to patients and their attitudes, behaviors to patients and patients’ relatives but most of these training sessions are not well-organized and staff usually come there to chat, they don’t pay much attention to the topics of the session. This leads to bad attitudes of some doctors to patients in hospitals. Many of them shout at patients and disregard patients. Besides, it is also noted that a small number of doctors receive extra money from patients that if patients don’t give them money, they will not behave and examine them well. These things make people have bad opinions about doctors and so make beer promoters - sensitive people - be afraid of going to doctors. (Vietnam, KI 3)

Another very important service that facilitates access to health care services for beer promoters is evening or weekend clinic hours. The beer promoters are often not able to go to the clinics during working hours, and many work late and are not willing to get up early to wait in a clinic to be seen. This Vietnamese beer promoter summarized her and her colleagues’ desires for health care institutions:

Of course, we always hope to have a centre with cheap price, convenience, the open time is from morning till midnight because sometimes it is difficult for us to arrange time for going to doctors. Besides, doctors must have good expertise and warm attitude to welcome us. (Vietnam, FG BP 2)

In addition to improving staff attitudes and after hours availability, several beer promoters commented on the availability of supplies and medication as an issue.

The hospital which is near my house does not have enough materials, but the far one demands more money. I just want them to have enough materials when we go. I don’t want to move to another hospital. (Cambodia, FG BP 4)

Finally, Cambodian and Laotian beer promoters noted that some NGO health care institutions provide incentives to encourage beer promoters to avail themselves of services. For example, one Cambodian NGO gives prizes to women who attend their clinic. A Laotian NGO provides beauty services and games for the beer promoters to make them feel more comfortable. These places encourage beer promoter clientele and thus facilitate their attendance for their health care needs as well.

Thus staff attitudes, clinic opening times, and medication availability can act barriers or a facilitators for beer promoters to access sexual and reproductive health care services. Another potential facilitator of service access is incentives to attract beer promoters. In addition to these institutional factors, however, the challenge of taking time off from work impacted on beer promoters access to health care services.

#### Work factors

One of the most common barriers to accessing reproductive health care services for beer promoters reported by both beer promoters and key informants was lack of time. Over one third agreed that they could not get time off of work to access health care (item 23). This varied from 25 percent for the Laotian beer promoters to 45 percent of those from Thailand and Vietnam. Many found that they were discouraged by their employers to take time away from work in order to seek health care. For example, this Cambodian beer promoter pointed out that the owner would cut their pay, and may not believe them if they requested time off to see a health care provider: When we want to have our health checked up, the company owner does not have time for us. If we want to go out, they will cut off our salary from 5 or 3 dollars. Sometimes we want to go to get the health care services, we ask for permission. But they think that we tell lies and we want to go somewhere else. (Cambodia BP FG1)

A Thai key informant noted that the working hours of beer promoters also prevent them from seeking health care: They don’t have enough time: they are working in the night time and wake up very late so they can’t go to the hospital. (Thai KI 4)

Not all beer promoters agreed that time was an issue for them. One Laotian beer promoter stated: Time is not a problem. We are allowed to go to health care services anytime we want to. (Laos BP FG5)

A key informant who also worked as a health care provider in Laos noted that this was not the case, however, for the beer promoters she treated: The beer promoters have a very limited time to visit my clinic for treatment in sexual and reproductive health as they spend much of their time working. (Laos KI 3)

In Vietnam, one key informant summarized: Beer promoters and sex workers are managed by their employers about time, if they go to health centers or hospitals, it will take them lots of time and this doesn’t please their employers. (Vietnam FG KI 1)

Thus in general, time was an important factor associated with work that limited beer promoters from all four countries to access sexual and reproductive health care services. There was not universal agreement about this, but for many beer promoters, taking time off of work for their health was not encouraged by their employers. Many also did not want to lose income by taking off time from work to seek health care services.

#### Personal factors

There were several personal factors that beer promoters from across the region experienced that affecting their willingness to seek sexual and reproductive health care services. These can be characterized as shyness and fears, lack of knowledge, and support from family and friends.

#### Shyness and fears

Women from all four countries stated that sometimes they avoided getting reproductive health care services because they were “shy”. Of the beer promoters surveyed, close to a third agreed that they avoided care because of shyness (item 18), though in Thailand the shy women were close to half the cohort at 46 percent. Over 22 percent of the beer promoters agreed they avoided being examined because they were afraid (item 21). In the focus groups, “shyness” appeared to have a variety of meanings including a fear of being examined, a fear of others seeing the beer promoter in the clinic and a fear of the equipment itself. Below are several examples of how the women describe their shyness and fears:

But some of us are shy and do not want to let the doctors to check us, even me for the first time. They are afraid to let the doctors to see their vagina. (Cambodia, FG BP 1)

Most women/girls are too shy to go to the hospital. They buy medicine from the pharmacy to take hoping to stop their pain without consulting a doctor. (Laos, FG BP 2)

Interviewer: Why do you think that women don’t go to see the doctor?

Respondent 1: Shy.

Respondent 2: I was afraid also. If some things happen, I cannot tolerate.

Respondent 3: I was afraid of the medical equipment. (Thailand, BP FG 4)

While a few of the key informants who worked as health care providers stated that the beer promoters who came to see them were comfortable sharing their health problems, this Vietnamese key informant noted that: They [beer promoters] usually hesitate going to big hospitals as they are afraid of seeing acquaintances there and afraid other people may recognize their identity, so they often go to private clinics or health centres which are far from their working place and they usually come there at the time they think there are a few customers. (Vietnam, FG KI 2)

Thus shyness and fears of examination and being recognized by others are common themes amongst the beer promoters in these four countries. Another significant personal factor affecting their access to reproductive heatlh care is lack of knowledge.

#### Lack of knowledge

Beer promoters lack knowledge of both their sexual and reproductive health needs and the services available to them. This is sometimes a barrier in accessing health care services. Key informants are particularly aware of this knowledge gap.

Most of the women come to this clinic because of sexually transmitted diseases; I think they should learn how to practice safe sex behaviors. They do not go out with their clients all the time, but they have their boyfriends and are not using condoms. Also they lack the knowledge about family planning. It is easy for them to get pregnant because they are still young and sexually active. (Thailand, KI 2)

I think barriers to sexual and reproductive health care services of beer promoters are their limited knowledge of sexual and reproductive health care. They also don’t know much about these services that hospitals provide. Due to limited knowledge, they are easily influenced by other beer promoters, and they can follow advice of their colleagues. They also may not know how to prevent diseases, especially sexually transmitted diseases. (Vietnam, KI 3)

#### Support of family and friends

Recommendations of friends was an important factor in choice of health care institution for close to 60 percent of the surveyed beer promoters (item 16). However, in the focus groups there was some discussion amongst the beer promoters about the cooperation of their partners and the support they received from their families when they were ill. Many did not inform their families about symptoms or seeking care unless they were very unwell. Families generally encouraged them to seek health care. Partners may encourage the woman to seek care while avoiding treatment themselves - unless the partner has many symptoms. The Cambodian beer promoter quoted below commented on the challenges beer promoters experience, due to the lack of cooperation from partners.

One problem, sometimes her family pushes her to see doctor but the partner did not go with her. When we have vaginal discharge, if we were treated and partner did not go for treatment, we can infect each other. So the problem is the partner did not want to see doctor. He said that he is lazy to see doctor, only ask for medicines. (Cambodia, FG BP 1)

On the other hand, friends can facilitate access to health care services by making recommendations of where to seek care, as the Thai beer promoter described:

Interviewer: Do your friends influence you to choose the hospital?

Respondent: Yes, they do. If they told this hospital is good service, we will go. (Thailand, FG BP 2)

Thus friends and family can act as a support to the beer promoter, recommending health care services and encouraging access to services. Sometimes family acts as a barrier to health for beer promoters; in particular, uncooperative male partners who choose not to be treated can negatively impact on the beer promoters’ health status.

### Limitations of the study

There are two major limitations to this research. The first limitation results from our choice of conducting an international participatory research project. Training beer promoters as research assistants, though extremely important, did not change the fact that they were in fact novice data collectors. As a result, some of the qualitative data lacked depth. Opportunities for probing focus group members to elicit more detail were missed. While we did conduct a training session with the research team prior to data collection, it was limited in time and by language barriers. The Canadian researchers did not speak any of the Asian languages of the study, nor did any of the research assistants speak English fluently, thus direct observation of the research assistants’ skills in conducting focus groups and surveys was not possible. In future research, we would budget for a longer training period where the investigators could model the data collection techniques for research assistants with accompanying translation.

Despite the limitations of data collection, we feel that the choice of beer promoters as research assistants was useful for two key reasons. Firstly, the research assistants were able to make the beer promoters feel more at ease, and be willing to share more personal details than academic researchers could on their own. Secondly, this participatory research allowed the beer promoter research assistants to develop more skills that would improve their chances of getting a better job in the future.

Another major limitation of the research was the sampling strategy of the survey. In all four countries, convenience sampling was used, which can result in a selection bias. While randomization would have been preferable in order to obtain findings that were generalizable to the entire population of beer promoters in each site, this was impossible to do. Randomization requires a complete census of the beer promoter population in each site and this was beyond the resources of this study. Indeed, a census would be a challenge to undertake given the diversity of workplaces, fluidity of this employment, and the marginalized status these migrant women.

## Discussion

Rural to urban migrant women workers in Southeast Asia are among the waves of migrant workers in the region who move to larger cities or across borders in response to shifts in the globalized economy [26,27]. In this new urban environment, young women are compelled to negotiate amongst competing demands and desires from family and their peer network that vie for their attention and financial resources [28]. It is within this context that we situate the experiences and responses of the migrant beer promoters who engaged in our study.

These data provide some interesting observations and comparisons about beer promoters working in these four Southeast Asian capitals. While generalizations about differences in beer promoters between countries cannot be made, overall trends should be noted. For example, the Thai beer promoters tended to be young and single and not supporting any other family members. Most of these women had employee health insurance. Thailand is the most well off of the four participating countries, thus these women were mainly supporting themselves alone, while many of the women from Cambodia, Laos, and Vietnam were supporting other family members. The Thai beer promoters were less likely to participate in sex work or drink with their clients. This may be because their beer company and restaurant employers did not permit this, or because they were working as beer promoters to supplement their income or support themselves as students and did not have to resort to sex work like many of the beer promoters from the other three countries.

The health behavior data confirms that beer promoters are at risk of unwanted pregnancies and sexually transmitted infections. Over one third partake in sexual relationships outside of a primary relationship and close to a half of the women participate in sex work at least yearly. More than half of the women have had an abortion. With the exception of the Thai sample, over half have had a suspected or known sexually transmitted infection in the past. With the exception of the Thai cohort once again, most are drinking at work and a sizable proportion are getting drunk as a result, putting them at risk of making unwise decisions about their sexual health (i.e. engaging in unsafe sexual practices). It should be noted that drinking at work is sometimes required for those beer promoters who work on commission, thus is a risk of the job itself. As noted earlier, differences between countries may be reflected by different expectations of the employers. The high prevalence of these behaviors indicates that beer promoters require access to quality sexual and reproductive health care services, as they are at risk of unwanted pregnancies and sexually transmitted infections including HIV/AIDS.

There was significant dissatisfaction with the current provision of health care services for beer promoters. In particular, cost of services, location and both environmental (long waits, lack of cleanliness and confidentiality) and service factors (health care provider attitudes) impact on access to health care services for beer promoters. External factors impacting on access to health care services include working hours: despite regulations in some countries, beer promoters often cannot get time off from work to get their health care needs met. Finally, personal factors such as shyness and fears, and lack of support from partners may prevent them from seeking care.

Such barriers to care are not unique to beer promoters in these four Southeast Asian capitals. Unmarried female migrant workers in China often do not access reproductive health care services because of social, psychologic and economic barriers [29]. Researchers have called for tailored interventions and more research on this large population of women [30].

Sex workers in Asia also face challenges accessing health care services. While population-based interventions to provide reproductive health care services are available, it is estimated that HIV prevention services in Asia are accessed by less than half of sex workers [31]. Like the beer promoters in this study, sex workers in Thailand report that cost, location, hours of operation, and friendly service are important issues to them, though perceived effectiveness of care was the strongest determinant of where they would access health care [32]. Similarly, Nepalese sex workers also have challenges accessing health care services due to inappropriate clinic opening hours, attitudes of the service providers, lack of confidentiality, fear of public exposure, and higher fees for the services [33]. Sex workers in Chennai, India experience personal, family and health care system barriers when accessing free HIV prevention services: stigma, discrimination and negative interactions with health care workers are particularly important issues affecting access to care for these women [34]. There are similar challenges with access to reproductive health care for sex workers in Afghanistan [35] and in Singapore, especially for those working free-lance outside of brothels [36]. Addressing stigma towards sex workers within society and amongst health care providers has been theorized as a necessary step to improve access to health care services for this vulnerable and diverse population of women [37], and would have a positive impact on beer promoters as a significant number of them also engage in sex work.

Table 6 lists potential barriers, and facilitators to improve access to sexual and reproductive health care services for these women. This table is intended to generate discussion to develop more specific recommendations for each community. One of the most important considerations at a policy level is to improve access to health care insurance. Either government or employer health care insurance, available from their community of residence not birth, would ensure that women have access to health care services when they need it. Other solutions require a more location-specific approach such as building clinics close to the work-place of beer promoters or providing mobile clinics that beer promoters can access easily. Improving access to health care institutions will require a recognition by the management of these institutions of the importance of serving this population of women and eliminating barriers preventing them from accessing service. Reducing waiting times for working women, ensuring space within the institution for confidential discussions and teaching health care providers the importance of confidentiality, keeping the clinic area clean, and particularly improving health care providers attitudes towards beer promoters - reducing the stigma they experience when accessing health care - are universal issues to be addressed by health care institutions in all four countries. Addressing these issues would improve health care access for all users, not just beer promoters. In addition to policy changes and institutional improvements, there is a need to enforce the human rights of these workers in having time off from work to access health care services as needed. Finally, public health education campaigns targeting migrant workers and their sexual partners may help to improve understanding of the need for and availability of health care services.

**Table 6:** Barriers and Facilitators to Improving Access to Reproductive Health Care Services for Beer Promoters

**Potential Barriers**	**Facilitators: Interventions to Improve Access**
INSTITUTIONAL FACTORS	
Cost	Provide free or low cost clinics.
	Provide health care insurance through employer or government plans, ensure migrants are included.
Location	Build institutions near where beer promoters live and work.
	Provide free transportation to health care institutions.
	Provide mobile clinics that visit locations where beer promoters work.
Waiting Times	Try to reduce waiting times by hiring more staff for busy periods and giving working women quicker access.
	Provide evening and weekend clinic hours.
Confidentiality	Ensure quiet spaces for confidential conversations and train staff about the importance of confidentiality.
Cleanliness	Stress cleanliness in institutions and have regular inspections by administration and/or government health inspectors.
Staff Attitudes	In-service training focusing on stigma against women who work in beer promotion and sex work.
	Surveys of clients to seek feedback on their experiences in the institutions and active incorporation of ideas to improve service.
Lack of Medication and Materials	Appropriately fund sexual and reproductive health institutions to have needed medications and materials.
	Facilitate referrals to higher levels of care as needed.
Institution does not attract beer promoter clients	Provide incentives to beer promoters such as prizes, food, social activities, etc.
WORK FACTORS	
Lack of Time	Provide evening and weekend clinic hours.
	Create and enforce government policies that require employers to provide access to health care services for beer promoters.
PERSONAL FACTORS	
Shyness and Fears	Education about importance of sexual and reproductive health care directed at beer promoters in workplace and media.
	Orientation of beer promoters to health care facilities and equipment by friendly staff. Women providers as needed for reproductive examinations.
Lack of Knowledge	Education and orientation as discussed above.
Support of Family and Friends	Education of male partners about the importance of treatment.
	Provide incentives to beer promoters to bring friends for assessment and treatment.

## Conclusions

Clearly, improving access to health care services for this population of women will require a multiple intervention approach, tackling factors both within and outside the health care system, at both institutional and personal levels. While the situation for beer promoters in each of these four Southeast Asian capitals has unique features, all of these women experience barriers to accessing sexual and reproductive health care services. It is our hope that local governments, employers and health care institutions will adopt some of these solutions to improve access and ultimately the sexual and reproductive health care status of these rural-to-urban migrant workers.

## Abbreviations

AIDS: Acquired Immune Deficiency Syndrome; HIV: Human Immundeficiency Virus; ILO: International Labour Organization; NGO: Non- governmental Organization; UNAIDS: Joint United Nations program on HIV/AIDS.

## Competing interests

The authors declare that they have no competing interests.

## Authors’ contributions

GW conceived of the research study, wrote the funding application, assisted with training and managing the research teams, analyzed the data and wrote the first draft of the paper. DS assisted with the funding application, training and data analysis and gave input into the paper. RS managed the Thai research team and data collection, and gave input into the funding application and paper. TCD managed the Vietnamese research team and data collection, and gave input into the funding application and paper. SK managed the Lao research team and data collection, and gave input into the funding application and paper. All authors read and approved the final manuscript.
